# The Impact of the First Wave of the COVID-19 Pandemic on University Staff Dietary Behaviours, Sleeping Patterns, and Well-Being: An International Comparison Study

**DOI:** 10.3390/ijerph20206941

**Published:** 2023-10-19

**Authors:** Fatemeh Rabiee Khan, Maher Abdelraheim Titi, Natalia Frankowska, Katarzyna Kowalczyk, Rasmieh Alziedan, Christine Yin-Kei Lau, Karolina Biernat, Kyle Gavin Brown

**Affiliations:** 1 College of Life Sciences, Faculty of Health, Education and Life Sciences, Birmingham City University, Birmingham B15 3TN, UK; 2 Quality Management Department, King Saud University Medical City, Riyadh P.O. Box 7805, Saudi Arabia; 3 Research Chair for Evidence-Based Health Care and Knowledge Translation, Deanship of Scientific Research, King Saud University, Riyadh P.O. Box 7805, Saudi Arabia; 4 SWPS University of Social Sciences and Humanities, 03-815 Warsaw, Poland; nfrankowska@swps.edu.pl; 5 The Global Public Health Network, 02-796 Warsaw, Poland; kasia@cityhealthinternational.org; 6 Cardiology Department, Medical College, King Fuad University, Riyadh P.O. Box 7805, Saudi Arabia; rasmieh.alzeidan@mail.bcu.ac.uk; 7 Jockey Club School of Public Health and Primary Care, The Chinese University of Hong Kong, Hong Kong, China; ykclau@cuhk.edu.hk; 8 Solihull Metropolitan Borough Council, Public Health Department, Council House, Solihull B91 9QS, UK; karolina.biernat@solihull.gov.uk; 9 College of Psychology, Faculty of Business, Law and Social Sciences, Birmingham City University, Birmingham B4 7BD, UK; kyle.g.brown0@gmail.com

**Keywords:** COVID-19, lockdown, dietary behaviour, sleeping patterns, well-being, university staff, international comparison

## Abstract

This study assessed the impact of the first wave of the COVID-19 pandemic on well-being by measuring the changes to food security, dietary behaviour, and sleeping patterns of university staff in England, Poland, Saudi Arabia, and China. Using a cross-sectional study design, participants in four universities in the respective countries were surveyed between June and July 2020. The mean age of the 902 participants was 42 years old and 67% were female. The findings indicate a reduction in emotionally driven food behaviour [t (901.00) = −20.87, *p* <  0.001], food acquisition location [t (901.00) = −51.55, *p* < 0.001], skipping meals [t (901.00) = −24, *p* < 0.001], and consumption of canned fruit and vegetables [t (901.00) = −10.18, *p* < 0.001]. However, home cooking [t (901.00) = 36.61, *p* < 0.001] and the food shopping experience [t (901.00) = 4.53, *p* <  0.001] markedly increased during lockdown. The participants had higher levels of well-being during the pandemic and experienced a significant increase in sleeping hours (*p* < 0.001). Increased age and sleeping hours were positively associated with overall well-being. Conversely, emotionally driven food behaviour (i.e., buying and eating more food out of boredom/fear or anxiety) and skipping meals decreased the overall well-being. Lockdown had beneficial effects on dietary behaviours, sleeping patterns, and well-being, but there were variations between countries.

## 1. Introduction

The spread of COVID-19 was swift and borderless, affecting millions of people around the globe [1]. The outbreak has had a significant impact on the health and well-being of the general population in all countries. The fear of contracting COVID-19 and the mandatory public measures, such as lockdowns and social distancing, impacted various lifestyle behaviours, such as physical activity, sleeping patterns, alcohol intake, and smoking [2,3]. The measures also affected dietary behaviours, such as public food acquisition, eating behaviour, food shopping, and household stockpiling [2]. Waiting a long time in queues, while maintaining social distancing, and anxiety about the possibility of contracting the virus possibly worsened well-being.

The impact of the pandemic on mental well-being appears to be linked to occupational characteristics during the COVID-19 pandemic [4]. For example, healthcare workers experienced poor well-being and psychological distress. In contrast, adult home workers reported lower depression and anxiety levels than those who continued to work in the workplace [5]. Staying and working at home could be linked to lower exposure to COVID-19 [6], but also allows for longer sleeping hours, regular meals, adherence to better personal hygiene, and less anxiety about exposure, which in turn relates to an overall feeling of well-being. A number of previous studies highlighted the significant importance of regular and high-quality meals for overall well-being [7,8], and illustrated the impact of sleep quality and both sleep hygiene awareness and practices on general well-being [9].

COVID-19 had a significant effect on the work of university staff, forcing them to work from home and change the way they provided services. This required a prompt response to develop new skills to teach in a virtual environment. Recent evidence suggests that about 30% of university staff experienced traumatic stress symptoms [10], anxiety, and depression [11]. How these worries affect their well-being warrants further study.

We acknowledge differences in the dietary and sleeping patterns in the general population in the four countries (England, Poland, Saudi Arabia, and China) [12,13,14,15], but the focus of this study was to investigate and compare the impact of lockdown on the dietary behaviours, sleeping patterns, and well-being of university staff. Except for China, where COVID-19 was detected in the autumn of 2019 and a strict lockdown was implemented at the end of January 2020, the pandemic scenario in Poland, England, and Saudi Arabia was similar. The first protective measures were implemented in March 2020 and included closing schools, university classes, offices, cultural institutions, cancelling mass events, and mandatory face coverings.

We assumed that the first wave of lockdown measures would have a significant impact on the dietary behaviours, sleeping patterns, and well-being of university staff. Therefore, the aim of this study was to explore the consequences of the first wave of the COVID-19 pandemic on university staff in four countries in relation to whether:

- Dietary behaviour, sleeping patterns, and well-being differed during the first wave of COVID-19 compared to pre-pandemic times;
- Dietary behaviour and sleeping were associated with well-being;
- Differences occurred between the countries involved in the study.

## 2. Materials and Methods

This study follows the guidelines in the Strengthening the Reporting of Observational Studies in Epidemiology (STROBE) statement [16].

### 2.1. Study Design and Settings

The study is a cross-sectional online survey. The target population was staff from three public and one private non-profit university across four countries: England, Poland, Saudi Arabia, and China. The universities were selected for a cross-cultural comparison based on the affiliations of the research team. This paper is part of a larger study, which focuses only on dietary behaviours, sleeping patterns, and well-being.

### 2.2. Data Collection and Procedure

Data was collected using the Qualtrics online survey software (Qualtrics, Provo, UT, USA). An anonymised email address was set up via the universities’ IT teams, and the questionnaire was sent to all the staff within each university. Permission to access was obtained from the relevant authorities in each university. In Poland and Saudi Arabia, the web survey link was also placed in the academic forum. A data sharing agreement was signed by each participating institution.

Online informed consent was obtained before the start of the questionnaire and was anonymised at the point of data collection. The multi-country research design was submitted by the principal research investigator in England and approval was granted by Birmingham City University’s (Faculty of Health, Education, and Life Sciences) ethics committee REF:7378/R(A)/2020/Apr. Other partner countries then used this approval and applied to their relevant ethics committee for approval. Our study protocol [17] provides further details regarding data protection. The questionnaire took a maximum of 15 min to complete.

### 2.3. Sampling Technique and Sample Size

A convenience sampling technique was used. All employees working in the selected universities were eligible and invited to participate. Because of the self-selected and non-probabilistic nature of the sample, the response rates were not quantifiable, as highlighted by the American Association for Public Opinion Research’s (AAPOR) reporting guidelines [18]. The estimated minimum sample size required to show effects was 178, calculated for a basic multiple regression (Section 3.3) assuming a medium effect size, an alpha of 0.05, and 10 predictors at 0.95 power. This calculation was carried out using GPower 3.1.

### 2.4. Questionnaire

The online questionnaire was adapted using two questionnaires developed by the United Nations Standing Committee on Nutrition (UNSCN) [19] and validated COVID-19 International Student Well-being Study [20]. The questionnaire consists of 11 domains: sociodemographic information (18 items), changes in dietary behaviours (25 items), weight changes (1 item), smoking habits (6 items), physical activity changes (2 items), alcohol consumption changes (2 items), sleep changes (1 item), well-being status during the pandemic (14 items), workload changes (5 items), COVID-19 diagnosis, symptoms, perceived worries (3 items), and health conditions (1 item). The questionnaire’s content validity was evaluated by a panel of experts on nutrition, public health, psychology, and statistics. In the case of Poland and Saudi Arabia, the questionnaires were translated into the respective native languages using appropriate guidelines [21]. Following this translation process, a rigorous examination was undertaken by experts in the field to ensure accuracy and cultural appropriateness.

Modifications were made following a pilot involving 10 participants in each country. The factor structure and reliability of the unvalidated nutrition items were assessed using factor analysis and McDonald’s omega, respectively [22]. Factor loadings lower than 0.3 were suppressed. One factor, “Leaving the house to shop for groceries” from the “Food acquisition location”, was removed to increase reliability. Changes in workload did not meet our threshold for reliability (omega = 0.6) and were, thus, excluded from further analysis (Figure S1, Tables S1 and S2 in the Supplementary Materials).

### 2.5. Variables and Data Measures

#### 2.5.1. Dietary Behaviours

The following variables were used to measure the changes in dietary behaviours (Box 1):

Box 1 **Changes in dietary behaviour Variables.**

▪ Changes in emotionally driven food behaviours (i.e., buying and eating more food out of boredom, fear, or anxiety) that were measured using six questions (on a 5-point scale, from “Definitely disagree” to “Definitely agree”).
▪ Changes in food acquisition location (i.e., eating outside the home) were measured using four questions (on a 5-point scale, from “decreased” to “increased”).
▪ Changes in food shopping experience were measured using four items (on a 5-point scale, from “Not at all” to “Very much”).
▪ Changes in fresh food consumption were measured using two questions (on a 5-point scale, from “Definitely don’t agree” to “Definitely agree”).
▪ Changes in consuming food reserves (i.e., eating more canned fruit and vegetables) were measured using four questions (on a 5-point scale, from “Definitely don’t agree” to “Definitely agree”).
▪ Changes in home cooking behaviour were measured using three items (on a 5-point scale, ranging from “decreased” to “increased”).
▪ Changes in skipping meals were assessed using two items (on a 5-point scale, from “Definitely don’t agree” to “Definitely agree”).

#### 2.5.2. Well-Being and Sleep

One item examined changes in sleeping hours before and during lockdown. Mental well-being during the pandemic was examined using 14 questions [20] on a 5-point Likert scale, ranging from “none” to “almost all the time”, and incorporating the eight-item abbreviated version of the Centre for Epidemiological Studies Depression Scale (CESD-8) [23,24] and an additional six items into an index on well-being.

### 2.6. Data Analysis

Data coding, cleaning, merging, and analysis were undertaken by a single author and checked by the team, to minimize errors and increase reliability. After descriptive analyses, the normality of the data was checked by using the Shapiro–Wilk test; *t*-tests were then carried out to examine perceived differences in the dietary behaviours and well-being before and during lockdown. Specifically, (1) one sample *t*-test was carried out on the single score measures to determine whether the scores differed from the scale midpoint (i.e., a test value of 3 on the 5-point scale), and paired *t*-tests were used for questions with separate items for before and during lockdown. (2) To ascertain the extent to which the combination of variables significantly predicted well-being, we applied a linear mixed model [25], with gender as a fixed factor and age, alongside each of the dietary behaviours entered as covariates. This was achieved using the GAMLj package in Jamovi (2021 v1.8.2) [26]. The participant’s country/university was entered as a random intercept to account for potential clustering. The model failed to converge when random slopes were included. For this analysis, scores assessing perceptions on sleeping hours before and during COVID-19 were combined to create a single difference, or “change” score (score during and before COVID-19), where higher scores mean an increase in the respective factor over time. A MANCOVA, accompanied by Pillai’s trace and follow-up ANCOVAs, were utilised to compare dietary behaviour scores across the respective collaborating universities and countries. It is worth mentioning that Pillai’s trace is a multivariate test statistic that is used in MANOVA (multivariate analysis of variance) and MANCOVA analysis. It assesses the overall significance of the differences between groups, while considering all the dependent variables collectively. A chi-square test for independence, with α= 0.05, was used to assess the categorical variables.

### 2.7. Missing Data Analysis

In the final sample, we observed a general trend of increased missing data as participants chronologically progressed through the study, ranging from 2 participants not answering a question on gender to 94 not completing the questions on the primary outcome (well-being). As most missing data occurred for the primary outcome, we carried out a multiple imputation, missing value analysis (in IBM Corp. Released 2020. IBM SPSS Statistics for Windows, Version 27.0. Armonk, NY, USA: IBM Corp) [27], using an expectation maximisation algorithm. This was carried out for the variables associated with our primary analyses of interest (dietary outcomes and well-being) to ensure there was sufficient power. Table 1 provides a breakdown of the missing data. Little’s MCAR test was not significant χ^2^ (58) = 64.655, *p* = 0.256, indicating that the data were missing completely at random. As a result, automatic multiple imputation (using a linear regression model) was carried out to fill in the missing values.

## 3. Results

### 3.1. Sociodemographic Characteristics of Study Population

A total number of 1597 university staff participated in the study. The participant’s data were included if they completed the demographics section and spent at least 5 min on the study. This resulted in a final sample of 902 (56.5%) participants, with a mean age of 42.05 (SD = 11.60). More than two-thirds of the participants in all countries, except Saudi Arabia, were female. In Saudi Arabia, the proportion of males and females was approximately similar (46.7% vs. 53.3%). Over two-thirds of the participants in all countries were teaching and/or research staff; except in the UK, where only 52% were teaching and/or research staff and the rest were in management and administration (Table 2).

### 3.2. Impact of COVID-19 on Dietary Behaviours, Sleeping Patterns, and Well-Being

The results show a significant change in all the outcomes assessed with a single item. COVID-19 appears to have had the largest impact on where people ate, with a perceived decrease in the food acquisition location (i.e., eating outside the home), with the largest effect size, followed by an increase in home cooking. The next largest effects correspond to a change in the food shopping experience. Significant decreases in skipping meals also had a large effect size, based on Cohen’s criteria (i.e., d > 0.8) [28]. Next, with moderate effect sizes, our participants experienced less emotionally driven food behaviours and greater well-being since the lockdown started. There were also small effects highlighting a slight decrease in the consumption of food reserves. The effect size corresponding to the increase in fresh food consumption was very small (Table 3). Paired sample *t*-tests (on items assessing perceptions both before and during lockdown) revealed that the average hours of sleep significantly increased (t (828) = −6.83, *p* < 0.001), from an average of 6.09 h (SD = 1.09) prior to lockdown, to 7.23 h (SD = 1.37) during lockdown.

### 3.3. Impact of Dietary Outcomes on Well-Being

The results from the linear mixed model showed an overall model fit (r^2^ = 0.27, AIC = 1655.06, BIC = 1799.91, LL = −845.55), with age, emotionally driven food behaviour, skipping meals, and change in sleeping hours, all significantly related to well-being after controlling for country (Table 4). Increased age (B = 0.01, t (871.29) = 3.59, *p* < 0.001) and longer sleeping time (B = 0.11, t (871.04) = 5.05, *p* < 0.001) were positively associated with overall well-being. Conversely, emotionally driven food behaviour (B = −0.14, t [871.56] = −5.33, *p* < 0.001) and skipping meals (B = 0.13, t (870.61) = −6.61, *p* < 0.001) decreased the overall well-being.

### 3.4. Impact of Sociodemographic Groups on Findings

A MANCOVA was conducted to determine whether each of the composites of dietary behaviours differed according to country, whilst controlling for participants’ age and gender.

The results from Pillai’s trace showed that country, gender, and age each had an impact on at least one of the composite food-related behaviours. In other words, these factors collectively contributed to the differences observed in participants’ food-related behaviours. The analyses revealed that country (V = 0.61, F (30, 2622) = 22.48, *p* < 0.001), gender (V = 0.03, F (10, 872) = 2.71, *p* = 0.003), and age (V = 0.03, F (10, 872) = 2.91, *p* = 0.001) each predicted at least one of the dietary behaviours, so follow-up ANCOVAs were conducted to compare each country’s responses in regard to the composite variables. Where omnibus tests are significant, the significant (Tukey adjusted) post-hoc tests are reported below, whereas the non-significant follow-ups are presented in Figure S2, in Supplementary Materials.

#### 3.4.1. Emotionally Driven Food Behaviour

**Figure 1:**
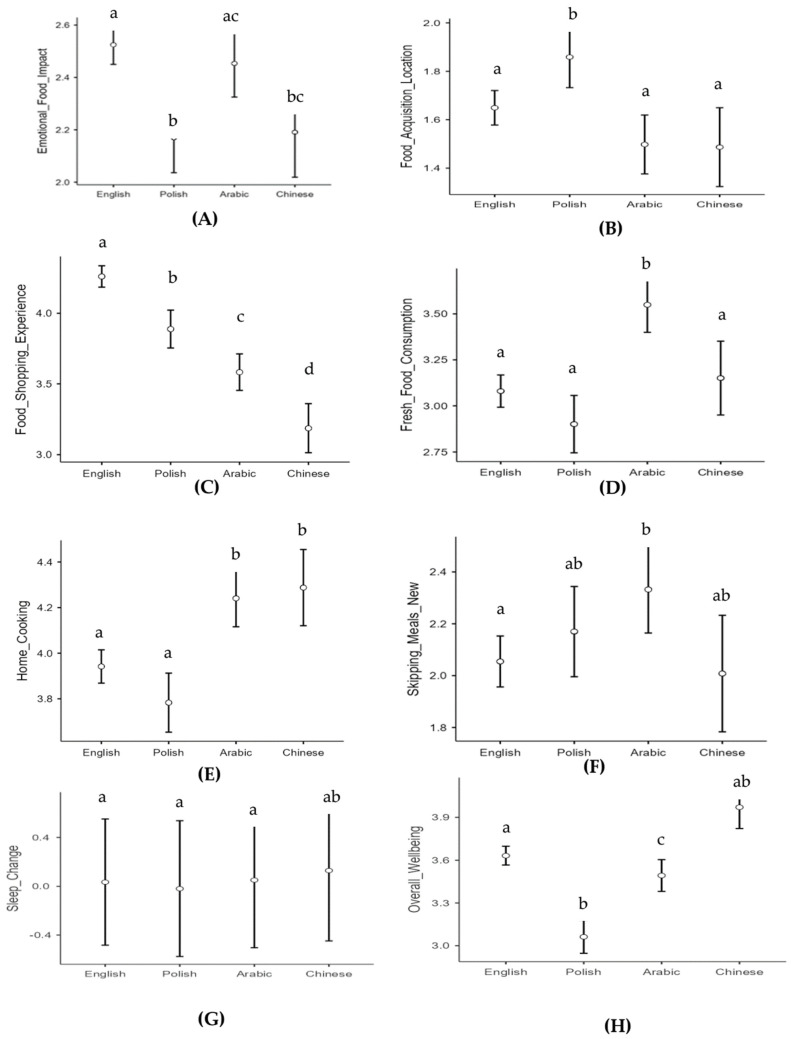
Difference between countries per measurement. Different letters denoted significant differences between countries.

Changes in emotionally driven food behaviour differed between participants across countries, F (3, 881) = 9.07, *p* < 0.001, ηp^2^ = 0.03, with participants based in England exhibiting more significant results than participants in Poland (t (881) = 4.51, *p* < 0.001, d = 0.43) and in China (t (881) = 3.48, *p* = 0.003, d = 0.40). Those in Saudi Arabia, similarly, had more emotionally driven food behaviours than individuals in Poland (t (881) = −3.02, *p* = 0.014, d = 0.34). Age was also positively related to changes in emotionally driven food behaviour, F (1, 881) = 8.66, *p* = 0.003, ηp^2^ = 0.01, but gender was not (Figure 1A).

#### 3.4.2. Food Acquisition Location

Whilst the analyses revealed an overall change in where the participants acquired food, there were also differences between the countries in this regard, F (3, 881) = 6.88, *p* < 0.001, ηp^2^ = 0.02. Participants from Poland perceived the greatest change, which was significantly higher than those in England (t (881) = 2.80, *p* = 0.03, d = 0.27), Saudi Arabia (t (881) = 4.05, *p* < 0.001, d = 0.46), and China (t (881) = 3.58, *p* = 0.002, d = 0.48). Neither gender nor age had any significant influence on food acquisition location (Figure 1B).

#### 3.4.3. Food Shopping Experience

Countries also significantly differed with respect to their experience whilst shopping, F (3, 881) = 56.03, *p* < 0.001, ηp^2^ = 0.16. All inter-country differences were significant, with those in England perceiving the greatest change to the experience relative to those in Poland (t (881) = 4.70, *p* < 0.001, d = 0.45), Saudi Arabia (t (881) = 8.83, *p* < 0.001, d = 0.82), and China (t (881) = 11.10, *p* < 0.001, d = 1.29). Gender and age did not significantly influence food shopping experience (Figure 1C).

#### 3.4.4. Fresh Food Consumption

Individuals across some countries differed in the extent to which they believed the lockdown had an impact on their fresh food consumption, F (3, 881) = 13.25, *p* < 0.001, ηp^2^ = 0.04. Those in Saudi Arabia experienced greater change, in comparison to those in China (t (881) = 3.12, *p* = 0.010, d = 0.41). The change in Saudi Arabia and China was greater than in both England (t (881) = 5.27, *p* < 0.001, d = 0.49) and Poland (t (881) = 5.90, *p* < 0.001, d = 0.67). Gender and age did not significantly influence fresh food consumption (Figure 1D).

#### 3.4.5. Consuming Food Reserves

There were no significant changes between countries in regard to their perceived consumption of food reserves, F (3, 881) = 0.71, *p* = 0.546, ηp^2^ = 0.00 (Figure S2, Tables S3–S17 in Supplementary Materials).

#### 3.4.6. Home Cooking

Countries differed in the extent to which they perceived themselves to be engaging in more home cooking during the pandemic, F (3, 881) = 13.17, *p* < 0.001, ηp^2^ = 0.04. Those in China had the highest scores, which were significantly higher than for those in England (t (881) = 3.70, *p* = 0.001, d = 0.43) and Poland (t (881) = 4.73, *p* < 0.001, d = 0.63), but did not significantly differ for individuals in Saudi Arabia. Similarly, those in Saudi Arabia had significantly higher scores than those in England (t (881) = 4.03, *p* < 0.001, d = 0.37) and Poland (t (881) = 5.00, *p* < 0.001, d = 0.57). Those in England and Poland did not significantly differ. As above, gender and age did not significantly influence home cooking (Figure 1E).

#### 3.4.7. Skipping Meals

Although respondents skipped fewer meals on average across the sample, there were some differences between countries, F (3, 881) = 3.01, *p* = 0.029, ηp^2^ = 0.01. Respondents from England (t (881) = 2.78, *p* = 0.028, d = 0.26) skipped meals comparatively less than those in Saudi Arabia, but there were no other differences. Gender, F (1, 881) = 1.34, *p* = 0.247, ηp^2^ = 0.00, did not affect skipping meals, but older individuals were more likely to skip meals, F (1, 881) = 5.86, *p* = 0.016, ηp^2^ = 0.01 (Figure 1F).

#### 3.4.8. Change in Sleep

The countries did not differ in how their sleep patterns changed during the pandemic, F (3, 879) = 0.24, *p* = 0.870, ηp^2^ = 0.00 (Figure 1G).

#### 3.4.9. Overall Well-Being

The increase in well-being experienced across the overall sample since the beginning of the pandemic was significantly different between countries, F (3, 881) = 35.66, *p* < 0.001, ηp^2^ = 0.11. Those living in China experienced significantly greater well-being compared to those in England (t (881) = 4.06, *p* < 0.001, d = 0.47), Saudi Arabia (t (881) = 5.03, *p* < 0.001, d = 0.67), and Poland (t (881) = 9.53, *p* < 0.001, d = 1.27), respectively. Gender did not affect well-being, but older individuals did experience greater well-being, F (1, 881) = 18.45, *p* < 0.001, ηp^2^ = 0.02 (Figure 1H).

## 4. Discussion

Overall, this study has identified that the first wave of COVID-19 had a significantly positive impact on university staff across the domains on dietary behaviours, increased sleeping hours, and well-being. Gender did not appear to be an influencing factor, but age was. Older participants slept longer hours and perceived a better feeling of well-being. There was no difference in the pattern of sleeping for the participants in any of the four countries studied. Significant variation was observed across countries in relation to certain dietary behaviours and well-being, which will be discussed further in detail.

Unlike other studies [14,29], our results reveal a significant reduction in emotionally driven food behaviour (i.e., buying and eating more food out of boredom/fear or anxiety). This positive finding amongst our participants could be due to a combination of age, having a higher educational level, a secure job and income, and individuals’ adaptive coping strategies [13]. Although the reduction occurred in all countries, England and Saudi Arabia showed the highest reduction rates. That could be related to their lower level of anxiety and better social support systems, which is outside the scope of this paper, but is an area that warrants further investigation.

Our results also suggest that the location for acquiring food (i.e., eating outside the home) has greatly diminished and home cooking markedly increased during lockdown. These results match the findings in other studies [13,30] and are likely related to forced confinement and closure of the hospitality sector. Another explanation is that cooking and baking became popular entertainment activities [13,31,32]. Furthermore, it could be related to a reduction in food deliveries due to fears about the spread of COVID-19 [33]. The variances in government policies and measures, response strategies, and social and economic status might explain the differences between the countries [34].

The food shopping experience was one of the earliest activities that was impacted by COVID-19 [30]. Our study showed significant changes in the shopping experience, such as restricted access to stores, physical distancing measures, and long queues [12,14,15,35]. Participants in England perceived the greatest change compared to those in Poland, Saudi Arabia, and China, respectively. All the countries introduced restrictions and rules on shopping for groceries [34,36]. It is also possible, though difficult to assess within this study, whether participants in England had a greater perception of food insecurity than their counterparts and, hence, perceived the greatest change.

Contrary to other studies [37], our research showed an increase in fresh food consumption (fruit and vegetables). This increase may be due to a perception of fresh food as a protective factor against COVID-19. The increase was highest among Saudi participants, which could be partly explained by an increase in public awareness towards healthy food consumption due to the introduction of various governmental healthy food strategies and their implementation in collaboration with key policymakers, private entities, and universities [38,39].

The consumption of food reserves decreased, but no significant differences were observed between the countries. Respondents from all countries perceived themselves to be eating more healthy food. The consumption of long shelf-life food, including canned fruit and vegetables, also decreased in all countries. Our findings echo previous research [32,40,41,42] and reflect the cultural belief that consuming healthy food, such as fresh vegetables, whole grains, and fresh meat, helps strengthen immunity and maintain good mental health [43].

Our findings also highlighted perceived greater well-being, indicating that healthy nutritional behaviour is associated with improved mental well-being among university staff. Previous research that studied the psycho-behavioural factors related to eating have reported perceived stress and anxiety to be associated with overeating [44], and also reported that skipping meals significantly increased the risk of mental distress, such as stress and depression and lower levels of psychological well-being [45]. The emotional brain networks and hormonal effects on eating behaviour, such as our food intake and eating motives, might explain our findings [46]. Thus, suggestions to encourage avoiding skipping meals should be emphasized to promote a balanced diet, which subsequently enhances psychological well-being. Our findings also demonstrate that boredom during lockdown is substantially important to address when considering a strategy to prevent disordered eating in this group.

Poor sleep quality and disrupted sleep have been linked to the COVID-19 pandemic in China [47], the UK [3], and Italy [11]. In contrast to previous research, our participants reported an increase in sleeping hours. Our findings are further supported by the suggestion that the quality of sleep appeared to get better as the lockdown continued [48]. In addition to adaptive coping strategies [13], home confinement likely permits more flexibility in sleep–wake timings and no requirement to wake-up early to commute to work [49].

Contrary to earlier findings [10,11], our results showed that university staff had higher levels of well-being during the first wave of lockdown. This could partly be related to the timing of this study. During June and July, academics and administrative staff usually have a lighter workload, the timing could have also enabled them to manage the transition to online working, which could, in turn, have had a positive impact on their well-being. Furthermore, staff might not have been worried about their academic progression, job security, and the financial implications. Similar effects were reported in a study on university staff in the Middle East and North Africa (MENA) region, where 67.4% of participants indicated a good level of mental well-being during the COVID-19 pandemic confinement [50].

Although well-being was high for all participants from the four countries, the Chinese participants recorded the highest level of well-being. A reasonable explanation for this finding could be the longer duration of the lockdown in China (4 months versus 2–3 months in the other three countries), and better adjustment by the Chinese participants compared with the Saudi, Polish, and British participants. Moreover, the greater resilience among those who tended to go outside more often, participated in more activities and benefitted from more social support [51], could well be responsible for this result.

In the current study, older participants had a greater level of overall well-being and longer sleeping hours when compared to younger participants. This further emphasises the important role of sleep on overall health, health-related behaviours, and well-being. An area on which public health strategies and health improvement campaigning should focus.

## 5. Strengths and Limitations

The strengths of this study lie in the comparison of a specific cohort (university staff) across countries, in addition to the assessment on how each factor is associated with well-being. The findings highlight similarities and differences amongst university staff and could be used as baseline data for future research. Our study was undertaken at the onset of the pandemic, during its initial wave, thereby capturing the initial adaptive reactions of a distinct demographic, namely academics and university staff. The specificity of this group primarily stems from the fact that an overwhelming majority possess university degrees and have a relatively higher socioeconomic status. It is worth noting that the longevity and sustainability of their responses throughout subsequent waves of the pandemic remain largely unknown.

Our study also has several limitations worth noting. One of them is a cross-sectional study design based on participant estimations on the extent to which their behaviour changed over lockdown, as opposed to a direct comparison of their behaviour before and during lockdown. This is a limitation to most similar studies, given the speed with which COVID-19 spread throughout the world. In our questionnaire, we asked participants to think about pre-COVID times. We relied on the respondents’ memory and their declarations about it, which is not reliable and is influenced by the time that has passed [52]. Moreover, records of the past in our memory are constantly being modified, which might affect the answers from our respondents [52]. Additionally, a high proportion of incomplete questionnaires from some countries may also have skewed our results towards countries with higher responses. However, low responses could not have been avoided at the time of data collection because different countries were at different stages of the pandemic and preparedness. Another reason for the low response rate could have been due to survey fatigue, as several questionnaires were concurrently administered by various academic groups, both within and externally, during our data collection period in the participating institutions. Utilising the Qualtrics software enabled us to analyse the time taken by participants to complete the survey. This approach proved instrumental in identifying false respondents [53], a circumstance that we encountered as well. Among the participants, there were instances where they merely clicked through the entire survey, without affording sufficient time to comprehend the content of the items. This phenomenon could point to various factors: perhaps they lacked motivation, especially since there was no compensation offered for participation, coupled with the survey being conducted during the summer holiday period. One additional significant limitation to this study is the absence of data regarding the income of the participants, which could exert a substantial influence on individuals’ access to food choices and their overall nutritional behaviours. Consequently, the lack of this information restricts our ability to comprehensively assess the potential impact of income, independent of other socioeconomic factors, on the observed dietary behaviours in our study. However, it has been well documented that differences in mental, physical, and educational outcomes are not fully accounted for by socioeconomic status [54,55,56]. Nevertheless, it is essential to acknowledge that our study did not incorporate controls for this factor.

## 6. Conclusions and Implications for Public Health

Overall, our participants experienced greater well-being during the first wave of lockdown, displayed less emotionally driven food behaviour, consumed less food with a long shelf-life, and consumed more healthy food than prior to the COVID-19 pandemic. They slept for longer and achieved higher scores for a better feeling of well-being. Our findings indicate how well-being, and the associated factors are likely to differ according to the country (and potentially associated demographics), even when working within the same occupational field. It also highlights how each of the factors impacted by COVID-19 do not necessarily relate to a significant change in well-being. We posit that several protective health effects yielded in our study are based on the occupation and the associated sociodemographic sample, which was comparatively unaffected by COVID-19, relative to other industries.

We could also assert that university staff in our study successfully adapted to a stressful lockdown that lasted 3–4 months, at the time of this study.

This result may indicate that some social/occupational groups may have certain predispositions that facilitate adaptation. This is further evidence of the likelihood that those with greater financial security cope better in adverse circumstances. Further research should consider a longer time perspective. The higher educational level, older age group (average 42 years old), and financial security of the participants could all have played a major role in their adjustment. It was pleasing to find out that, despite huge changes to their working conditions and environment, working from home had a positive role in the longer sleep experienced by participants, which in turn had an impact on their well-being. We assume that, unlike other population groups [57] who suffered from social isolation and financial insecurity, our participants’ social circumstances enabled them to cope better and adjust with the changes imposed. These findings may be transferable to other occupations in similar epidemics, both nationally and globally, and warrant further investigation. Our findings, therefore, serve as a basis for the public health response to similar future pandemics, by highlighting the need to redress social and economic inequality and design interventions to enable resilience and coping mechanisms for improved health and well-being.

In summary, we believe this international comparison study provides new knowledge during an unprecedented global pandemic and provides baseline data for the research community, practitioners, and policymakers. Learning from this experience will also enable practitioners and policymakers to plan appropriate initiatives to improve the public’s health during the current situation, as well as similar future global crises.

## Figures and Tables

**Table 1:** Descriptive statistics derived from missing data analysis.

Variables	N	Mean	Std. Deviation	Missing N (%)	No. of Extremes Low
Age	887	42.05	11.559	15 (1.7)	2
Emotionally driven food behaviour	856	2.4068	0.84017	46 (5.1)	0
Food acquisition location	866	1.6459	0.79195	36 (4.0)	0
Food shopping experience	848	3.9567	0.91336	54 (6.0)	23
Fresh food consumption	857	3.1289	0.97006	45 (5.0)	0
Consuming food reserves	857	2.8054	0.56850	45 (5.0)	0
Home cooking	866	3.9913	0.81580	36 (4.0)	18
Skipping meals	857	2.1219	1.09267	45 (5.0)	0
Change in sleep pattern before	829	6.9180	1.08816	73 (8.1)	4
Sleep pattern during	829	7.2292	1.36664	73 (8.1)	7
Overall well-being	808	3.5380	0.76316	94 (10.4)	1
Country	902			0 (0.0)	
Gender	900			2 (0.2)	

**Table 2:** Sociodemographic characteristics.

Sociodemographic Characteristics *		UK	Saudi Arabia	China	Poland	*p*-Value
**Gender**	Male	152 (31.5%)	78 (46.7%)	20 (23.3%)	32 (20.9%)	<0.001
	Female	330 (68.5%)	89 (53.3%)	66 (76.7%)	121 (79.1%)	
**Occupation**	Teaching staff	143 (30.8%)	48 (30.4%)	15 (18.1%)	29 (19.5%)	<0.001
	Research staff	22 (4.7%)	27 (17.1%)	20 (24.1%)	24 (16.1%)	
	Both teaching and research	76 (16.4%)	50 (31.6%)	38 (45.8%)	85 (57%)	
	Management and administration	223 (48.1%)	33 (20.9%)	10 (12%)	11 (7.4%)	

* Groups with low frequencies were excluded from gender (i.e., non-binary (n = 2) and prefer not to say (n = 12)) and occupation (i.e., IT (n = 23) and service staff (n = 19)), in order not to affect the validity of the chi-square test.

**Table 3:** Breakdown *t*-test results for nutrition composite variables and well-being.

Variables	N	Statistic	df	*p*	Mean Difference	SD	Effect Size (d)
Emotionally driven food behaviour	902	−20.87	901.00	<0.001	−0.58	0.84	−0.69
Food acquisition location	902	−51.55	901.00	<0.001	−1.36	0.79	−1.72
Food shopping experience	902	31.70	901.00	<0.001	0.96	0.91	1.06
Fresh food consumption	902	4.53	901.00	<0.001	0.15	0.98	0.15
Consuming food reserves	902	−10.18	901.00	<0.001	−0.19	0.57	−0.34
Home cooking	902	36.61	901.00	<0.001	1.00	0.82	1.22
Skipping meals	902	−24.32	901.00	<0.001	−0.88	1.08	−0.81
Overall well-being	902	21.11	901.00	<0.001	0.54	0.77	0.70

Note: H_a_ μ ≠ 3.

**Table 4:** Impact of Dietary Outcomes on Well-Being-linear mixed model.

Variables	F	Num df	Den df	*p*
Age	12.85	1	871.29	<0.001
Gender	0.97	3	870.47	0.407
Emotionally driven food behaviour	28.36	1	871.56	<0.001
Food acquisition location	0.68	1	870.42	0.411
Food shopping experience	1.16	1	872.83	0.281
Fresh food consumption	0.00	1	870.94	0.970
Consuming food reserves	0.56	1	870.65	0.454
Home cooking	0.74	1	871.78	0.391
Skipping meals	43.63	1	870.61	<0.001
Change in sleep	25.49	1	871.04	<0.001

Note: Satterthwaite method for degrees of freedom.

## Data Availability

All relevant data are within the manuscript and its supporting information files.

## Supplementary Materials

The following supporting information can be downloaded at: https://www.mdpi.com/article/10.3390/ijerph20206941/s1, Refs. [1,20,22,27] are cited in Supplementary Materials. Figure S1: Scree plot indicating Eigenvalues derived from the principal component analysis; Figure S2: Error bar charts containing estimated marginal means (95% CI) for the composite variables by country; Table S1: Composite variable reliability; Table S2: Pattern matrix following an oblique rotation of the UNSCN nutrition questions; Table S3: Results of the ANCOVA analysis examining Emotional Food Impact by country, controlling for age and gender; Table S4: Results of the Tukey adjusted post-hoc comparisons analysis examining Emotional Food Impact by country, controlling for age and gender; Table S5: Results of the ANCOVA analysis examining Food Acquisition Location by country, controlling for age and gender; Table S6: Results of the Tukey adjusted post-hoc comparisons analysis examining Food Acquisition Location by country, controlling for age and gender; Table S7: Results of the ANCOVA analysis examining Food Shopping Experience by country, controlling for age and gender; Table S8: Results of the Tukey adjusted post-hoc comparisons analysis examining Food Shopping Experience by country, controlling for age and gender; Table S9: Results of the ANCOVA analysis examining Fresh Food Consumption by country, controlling for age and gender; Table S10: Results of the Tukey adjusted post-hoc comparisons analysis examining Fresh Food Consumption by country, controlling for age and gender; Table S11: Results of the ANCOVA analysis examining Home Cooking by country, controlling for age and gender; Table S12: Results of the Tukey adjusted post-hoc comparisons analysis examining Fresh Food Consumption by country, controlling for age and gender; Table S13: Results of the ANCOVA analysis examining Skipping Meals by country, controlling for age and gender; Table S14: Results of the Tukey adjusted post-hoc comparisons analysis examining Skipping Meals by country, control-ling for age and gender; Table S15: Results of the ANCOVA analysis examining Consuming Food Reserves by country, controlling for age and gender; Table S16: Results of the ANCOVA analysis examining Overall Well-being by country, controlling for age and gender; Table S17: Results of the Tukey adjusted post-hoc comparisons analysis examining Overall Well-being by country, controlling for age and gender.
