# Supplementary material for: The Impact of the First Wave of the COVID-19 Pandemic on University Staff Dietary Behaviours, Sleeping Patterns, and Well-Being: An International Comparison Study
Source: Int J Environ Res Public Health. 2023 Oct 19;20(20):6941. doi: 10.3390/ijerph20206941 (PMC10606338; doi:10.3390/ijerph20206941)
Supplement: Supplementary file 1 [file ijerph-20-06941-s001.zip › ijerph-2378558-supplementary.pdf]

Online Supplementary materials

Supplementary material A – Composite variable reliability

Table S1- Composite variable reliability

Factor	Cronbach's Alpha	McDonald's Omega	Items/Questions	Alpha if question dropped	Omega if dropped
"Emotionally driven food behaviour"	0.83	0.83	I eat more sweets (cakes/chocolate)	0.81	0.81
			My body weight (increased…Decreased)	0.84	0.84
			I buy more food out of fear or anxiety	0.81	0.81
			I eat more out of fear or anxiety	0.78	0.78
			I buy more food out of boredom	0.78	0.78
			I eat more food out of boredom	0.77	0.77
"Food acquisition location"	0.45	0.59	Leaving the house to shop for groceries	0.58	0.69*
			Eating out (e.g., restaurants/cafeteria/fast food)	0.22	0.44
			Eating at someone else's place (e.g., family, friends)	0.22	0.44
			Ordering take-away or fast-food meals with delivery	0.43	0.65
"Food shopping experience"	0.79	0.81	Restricted access to stores (e.g., one in one out queuing systems)	0.76	0.8
			Physical distancing measures in stores (e.g., protective screens/barriers at cashiers)	0.7	0.72
			Information about responsible purchasing in stores (e.g., signage about only purchasing what is needed / not stockpiling etc.)	0.7	0.72
			Physical distancing information in stores (e.g., signage or queuing markers on the floor)	0.8	0.82
"Fresh food consumption"	0.84	0.84	I eat more vegetables	0.71	-
			I eat more fresh fruits	0.75	-
"Consuming food reserves	0.62	0.62	I eat more canned fruits/ vegetables	0.49	0.5
			I eat more ready to eat canned/frozen food	0.48	0.49
			I have stockpiled more food than usual	0.57	0.59
			I waste more food than usual	0.61	0.63
			I rely more on any alternative sources of food during this time (e.g., your own/community food production, wild food harvesting)	0.56	0.59
"Home cooking"	0.65	0.65	Cooking at home	0.47	-
			Baking at home (e.g., bread, rolls, cakes)	0.5	-
"Skipping meals"	0.88	0.88	I skip meals because I cannot be bothered to eat	0.8	-
			I skip meals for other reasons (for example lack of motivation to prepare food)	0.79	-
"Well-Being"	0.9	0.9	you felt depressed	0.89	0.89
			you felt that everything you did was an effort	0.89	0.89
			your sleep was restless	0.89	0.9
			you were happy	0.9	0.9
			you felt lonely	0.89	0.89
			you enjoyed life	0.9	0.9
			you felt sad	0.89	0.89
			you could not get going	0.89	0.9
			you were bored	0.9	0.9
			you were frustrated with things in general	0.89	0.89

			you felt anxious	0.89	0.89
			you felt calm and peaceful	0.9	0.9
			you lacked companionship	0.89	0.9
			felt isolated from others	0.89	0.9

* Reliability following item removal

One factor required removal of items to increase reliability: *"Leaving the house to shop for groceries"* from the "Food acquisition location" factor. Change in workload did not meet our threshold for reliability (omega = 0.6) [22] and were thus excluded from further analysis.

Supplementary material B – Factor Analysis

A factor analysis (utilising an oblique rotation) was carried out on the 25 items derived from the United Nations Standing Committee on Nutrition (UNSCN) questionnaire [1]. Other questions were not included (see Table 1) as they had been validated in other studies[20]. Kaiser-Meyer-Olkin (KMO) values were deemed "good", supporting the adequacy of the sample[27], above the acceptable limit of 0.5. Correlations between items were deemed sufficient, as indicated by a significant test of sphericity χ^2 (300) = 6831.246, p < .001. The analysis revealed seven factors with Eigenvalues greater than 1, making up 62.25% of the variance. This was utilised as the scree plot (Figure 1) showed ambiguity (i.e. 2 5, or 7 factors) regarding the number of factors to be extracted. It is important to mention components that have eigen values less than one were excluded.

Figure S1: Scree plot indicating Eigen values derived from the principal component analysis.

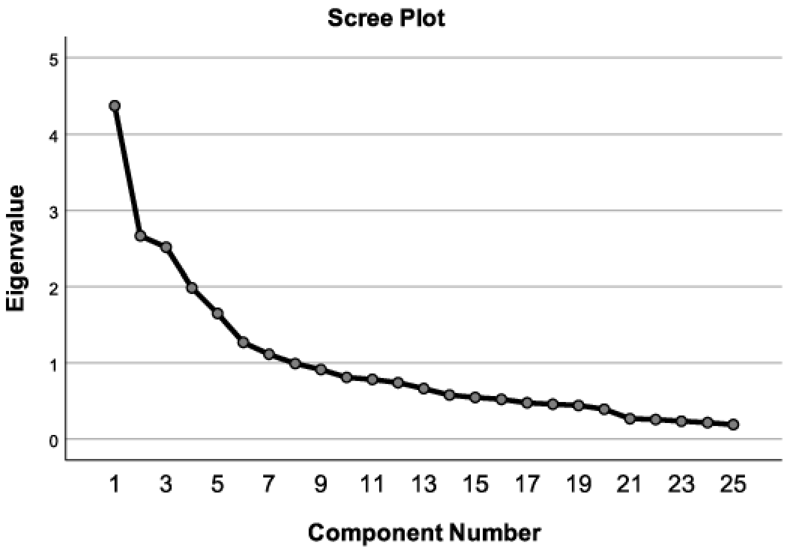

Loading of items to factors is highlighted in the pattern matrix below (table 1). Ambiguity regarding loading of the item "I have stockpiled more food than usual" was resolved by referring to the structure matrix, with which it loaded with items relating to food storage and waste (r = 0.51), as opposed to baking and cooking at home (r = 0.45) in the pattern matrix. All other loadings were consistent across the matrices.

Factors were interpreted as follows (table 1)

- Change in emotionally driven food behaviour.
- Change in food acquisition location.
- Change in food shopping experience.
- Change in fresh food consumption.
- Change in consuming food reserves.
- Change in home cooking.
- Change in skipping meals.

Table S2: Pattern matrix following an oblique rotation of the UNSCN nutrition questions.

	Component						
	1	2	3	4	5	6	7
I eat more food out of boredom	.823	.101	.014	.038	.095	.054	-.055
I buy more food out of boredom	.781	-.002	.061	.117	.185	.008	.037
I eat more food out of fear or anxiety	.731	.017	.056	.278	.148	-.025	.089
I eat more sweets (cakes/chocolate)	.698	.105	-.009	.116	-.050	-.006	-.070
During the lockdown my body weight:	.580	.018	.015	-.072	-.100	-.054	-.021
I buy more food out of fear or anxiety	.596	-.012	.074	.314	.105	.006	.256
Eating out (e.g., restaurants/cafeteria/fast food)	.023	.028	.893	.028	.044	-.033	-.081
Eating at someone else's place (e.g., family, friends)	-.038	.035	.871	.027	.050	-.080	-.056
Leaving the house to shop for groceries	.110	.058	.658	-.053	.007	.116	-.066
Ordering take-away or fast-food meals with delivery	.067	.010	.400	.192	-.009	-.087	-.365
Physical distancing measures in stores (e.g., protective screens/barriers at cashiers)	-.005	.860	.025	-.029	.006	.007	.021
Physical distancing information in stores (e.g., signage or queuing markers on the floor)	.012	.859	.007	-.016	.045	.011	.031
Restricted access to stores (e.g., one in one out queuing systems)	.059	.748	.033	.053	-.003	-.064	.002
Information about responsible purchasing in stores (e.g., signage about only purchasing what is needed / not stockpiling etc.)	.105	.681	.057	.046	.010	.030	-.004
I eat more fresh fruits	-.019	-.007	-.005	.046	.002	.911	.069
I eat more vegetables	-.004	-.015	-.010	.052	.012	.901	.152
I skip meals for other reasons (for example lack of motivation to prepare food)	.106	.039	.039	.144	.918	-.008	-.028
I skip meals because I cannot be bothered to eat	.103	.026	.054	.152	.913	.038	-.051
I eat more canned fruits/ vegetables	.036	.093	-.015	.787	.030	.141	-.114
I eat more ready to eat canned/frozen food	.235	.050	.045	.702	.114	-.087	-.040
I rely more on any alternative sources of food during this time (e.g. your own/community food production, wild food harvesting)	-.034	.015	-.008	.402	.122	.232	.229
I waste more food than usual	.294	-.092	.005	.450	.195	-.033	-.090
Baking at home (e.g., bread, rolls, cakes)	-.006	.037	-.062	.002	-.030	.079	.771
Cooking at home	.035	.022	-.209	.003	-.056	.133	.749
I have stockpiled more food than usual	.224	-.038	.087	.504	-.027	-.062	.430

** Factor loadings lower than 0.3 have been suppressed*

"Extraction Method: Principal Component Analysis.
Rotation Method: Varimax with Kaiser Normalization."

a. Rotation converged in 6 iterations.

Supplementary material C

Figure S2: Error bar charts containing estimated marginal means (95% CI) for the composite variables by country.

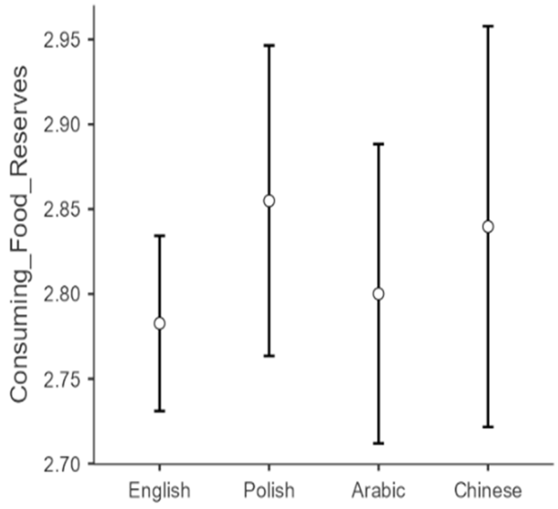

S2-A

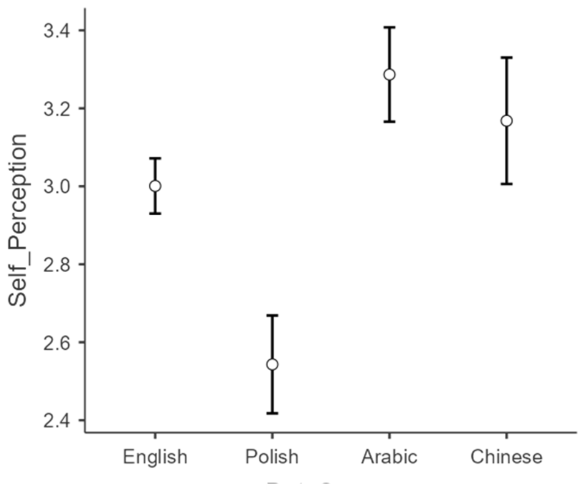

S2-B

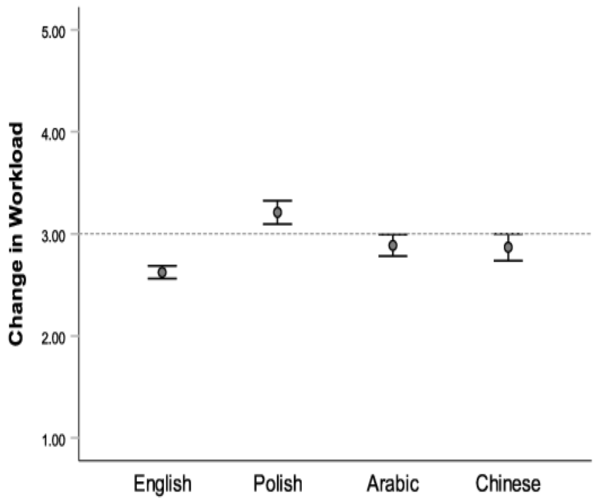

S2-C

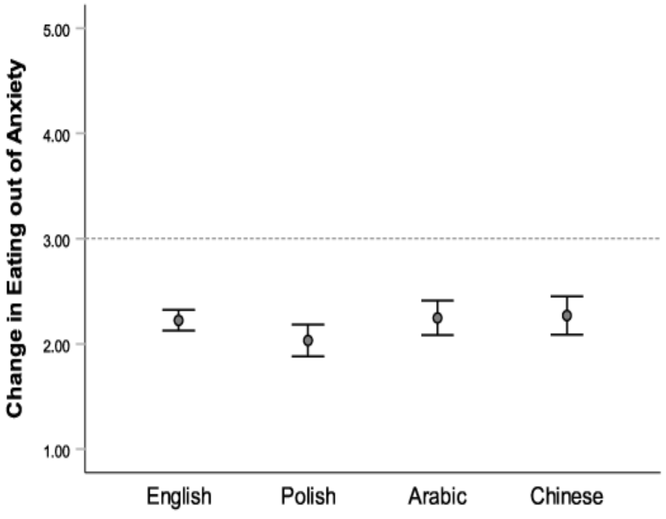

S2-D

Table S3: Results of the ANCOVA analysis examining Emotional Food Impact by country, controlling for age and gender

	Sum of Squares	df	Mean Square	F	p	η^2p
Overall model	26.58	5	5.32	6.58	< .001	
Gender	2.08	1	2.08	3.05	0.081	0.00
age	5.91	1	5.91	8.66	0.003	0.01
Country	18.59	3	6.20	9.07	< .001	0.03
Residuals	601.75	881	0.68			

Table S4: Results of the Tukey adjusted post-hoc comparisons analysis examining Emotional Food Impact by country, controlling for age and gender

Comparison								
**Country**		**Country**	**Mean Difference**	**SE**	**df**	**t**	**p_{tukey}**	**Cohen's d**
English	-	Polish	0.36	0.08	881.00	4.51	< .001	0.43
	-	Arabic	0.07	0.08	881.00	0.94	0.785	0.09
	-	Chinese	0.33	0.10	881.00	3.48	0.003	0.40
Polish	-	Arabic	-0.28	0.09	881.00	-3.02	0.014	-0.34
	-	Chinese	-0.02	0.11	881.00	-0.20	0.997	-0.03
Arabic	-	Chinese	0.26	0.11	881.00	2.40	0.078	0.32

Note. Comparisons are based on estimated marginal means.

Table S5: Results of the ANCOVA analysis examining Food Acquisition Location by country, controlling for age and gender

	Sum of Squares	df	Mean Square	F	p	η²p
Overall model	14.91	5	2.98	5.27	< .001	
Gender	0.19	1	0.19	0.31	0.575	0.00
age	2.08	1	2.08	3.39	0.066	0.00
Country	12.64	3	4.21	6.88	< .001	0.02
Residuals	539.40	881	0.61			

Table S6: Results of the Tukey adjusted Post-Hoc comparisons analysis examining Food Acquisition Location by country, controlling for age and gender

**Comparison**								
**Country**		**Country**	**Mean Difference**	**SE**	**df**	**t**	p_{tukey}	**Cohen's d**
English	-	Polish	-0.21	0.07	881.00	-2.80	0.027	-0.27
	-	Arabic	0.15	0.07	881.00	2.10	0.155	0.19
	-	Chinese	0.16	0.09	881.00	1.79	0.280	0.21
Polish	-	Arabic	0.36	0.09	881.00	4.05	< .001	0.46
	-	Chinese	0.37	0.10	881.00	3.58	0.002	0.48
Arabic	-	Chinese	0.01	0.10	881.00	0.11	1.000	0.01

Note. Comparisons are based on estimated marginal means

Table S7: Results of the ANCOVA analysis examining Food Shopping Experience by country, controlling for age and gender

	Sum of Squares	df	Mean Square	F	p	η²p
Overall model	116.94	5	23.39	36.47	< .001	

Table S7: Results of the ANCOVA analysis examining Food Shopping Experience by country, controlling for age and gender

	Sum of Squares	df	Mean Square	F	p	η^2p
Gender	0.07	1	0.07	0.11	0.742	0.00
age	0.69	1	0.69	1.00	0.317	0.00
Country	116.17	3	38.72	56.03	< .001	0.16
Residuals	608.85	881	0.69			

Table S8: Results of the Tukey adjusted Post-Hoc comparisons analysis examining Food Shopping Experience by country, controlling for age and gender

**Comparison**								
**Country**		**Country**	**Mean Difference**	**SE**	**df**	**t**	p_{tukey}	**Cohen's d**
English	-	Polish	0.37	0.08	881.00	4.70	< .001	0.45
	-	Arabic	0.68	0.08	881.00	8.83	< .001	0.82
	-	Chinese	1.07	0.10	881.00	11.10	< .001	1.29
Polish	-	Arabic	0.31	0.09	881.00	3.22	0.007	0.37
	-	Chinese	0.70	0.11	881.00	6.35	< .001	0.84
Arabic	-	Chinese	0.40	0.11	881.00	3.59	0.002	0.48

Note. Comparisons are based on estimated marginal means

Table S9: Results of the ANCOVA analysis examining Fresh Food Consumption by country, controlling for age and gender

	**Sum of Squares**	**df**	**Mean Square**	**F**	**p**	**η²p**
Overall model	37.07	5	7.41	8.30	< .001	
Gender	0.05	1	0.05	0.06	0.814	0.00
age	0.06	1	0.06	0.07	0.798	0.00
Country	36.96	3	12.32	13.25	< .001	0.04
Residuals	818.81	881	0.93			

Table S10: Results of the Tukey adjusted post-hoc comparisons analysis examining Fresh Food Consumption by country, controlling for age and gender

**Comparison**								
**Country**		**Country**	**Mean Difference**	**SE**	**df**	**t**	**p_{tukey}**	**Cohen's d**
English	-	Polish	0.18	0.09	881.00	1.95	0.210	0.19
	-	Arabic	-0.47	0.09	881.00	-5.27	< .001	-0.49
	-	Chinese	-0.07	0.11	881.00	-0.63	0.922	-0.07
Polish	-	Arabic	-0.65	0.11	881.00	-5.90	< .001	-0.67
	-	Chinese	-0.25	0.13	881.00	-1.95	0.208	-0.26
Arabic	-	Chinese	0.40	0.13	881.00	3.12	0.010	0.41

Note. Comparisons are based on estimated marginal means

Table S11: Results of the ANCOVA analysis examining Home Cooking by country, controlling for age and gender

	Sum of Squares	df	Mean Square	F	p	η^2p
Overall model	26.62	5	5.32	8.02	< .001	
Gender	1.12	1	1.12	1.74	0.188	0.00
age	0.03	1	0.03	0.04	0.834	0.00
Country	25.47	3	8.49	13.17	< .001	0.04
Residuals	567.91	881	0.64			

Table S12: Results of the Tukey adjusted post-hoc comparisons analysis examining Fresh Food Consumption by country, controlling for age and gender

Comparison								
Country		Country	Mean Difference	SE	df	t	p_{tukey}	Cohen's d
English	-	Polish	0.16	0.08	881.00	2.07	0.163	0.20
	-	Arabic	-0.30	0.07	881.00	-4.03	< .001	-0.37
	-	Chinese	-0.35	0.09	881.00	-3.70	0.001	-0.43
Polish	-	Arabic	-0.46	0.09	881.00	-5.00	< .001	-0.57
	-	Chinese	-0.50	0.11	881.00	-4.73	< .001	-0.63
Arabic	-	Chinese	-0.05	0.11	881.00	-0.44	0.971	-0.06

Note. Comparisons are based on estimated marginal means

Table S13: Results of the ANCOVA analysis examining Skipping Meals by country, controlling for age and gender

	Sum of Squares	df	Mean Square	F	p	η^2p
Overall model	18.93	5	3.79	3.78	0.002	
Gender	1.56	1	1.56	1.34	0.247	0.00
age	6.84	1	6.84	5.86	0.016	0.01
Country	10.53	3	3.51	3.01	0.029	0.01
Residuals	1026.98	881	1.17			

Table S14: Results of the Tukey adjusted post-hoc comparisons analysis examining Skipping Meals by country, controlling for age and gender

Comparison								
Country		Country	Mean Difference	SE	df	t	p_{tukey}	Cohen's d
English	-	Polish	-0.12	0.10	881.00	-1.12	0.678	-0.11
	-	Arabic	-0.28	0.10	881.00	-2.78	0.028	-0.26
	-	Chinese	0.05	0.13	881.00	0.37	0.983	0.04
Polish	-	Arabic	-0.16	0.12	881.00	-1.32	0.550	-0.15
	-	Chinese	0.16	0.14	881.00	1.13	0.672	0.15
Arabic	-	Chinese	0.32	0.14	881.00	2.27	0.107	0.30

Note. Comparisons are based on estimated marginal means

Table S15: Results of the ANCOVA analysis examining Consuming Food Reserves by country, controlling for age and gender

	Sum of Squares	df	Mean Square	F	p	η²p
Gender	2.18	1	2.18	6.76	0.009	0.01
age	0.87	1	0.87	2.69	0.101	0.00
Country	0.69	3	0.23	0.71	0.546	0.00
Residuals	283.87	881	0.32			

Table S16: Results of the ANCOVA analysis examining Overall Well-being by country, controlling for age and gender

	Sum of Squares	df	Mean Square	F	p	η²p
Overall model	64.67	5	12.93	28.37	< .001	
Gender	0.10	1	0.10	0.19	0.665	0.00
age	9.50	1	9.50	18.45	< .001	0.02
Country	55.07	3	18.36	35.66	< .001	0.11
Residuals	453.51	881	0.51			

Table S17: Results of the Tukey adjusted post-hoc comparisons analysis examining Overall Well-being by country, controlling for age and gender

Comparison								
**Country**		**Country**	**Mean Difference**	**SE**	**df**	**t**	**p_{tukey}**	**Cohen's d**
English	-	Polish	0.57	0.07	881.00	8.32	< .001	0.79
	-	Arabic	0.14	0.07	881.00	2.10	0.154	0.19
	-	Chinese	-0.34	0.08	881.00	-4.06	< .001	-0.47
Polish	-	Arabic	-0.43	0.08	881.00	-5.27	< .001	-0.60
	-	Chinese	-0.91	0.10	881.00	-9.53	< .001	-1.27
Arabic	-	Chinese	-0.48	0.10	881.00	-5.03	< .001	-0.67

Note. Comparisons are based on estimated marginal means
